# Adaptation to Sodium Hypochlorite and Potassium Permanganate May Lead to Their Ineffectiveness Against *Candida albicans*

**DOI:** 10.3390/ph17111544

**Published:** 2024-11-17

**Authors:** Tomasz M. Karpiński, Marzena Korbecka-Paczkowska, Marcin Ożarowski, Donald Włodkowic, Marzena Liliana Wyganowska, Agnieszka Seremak-Mrozikiewicz, Judyta Cielecka-Piontek

**Affiliations:** 1 Chair and Department of Medical Microbiology, Poznań University of Medical Sciences, Rokietnicka 10, 60-806 Poznań, Poland; mkorbecka@wp.pl; 2 Medi Pharm, os. Konstytucji 3 Maja 14/2, 63-200 Jarocin, Poland; 3 Department of Biotechnology, Institute of Natural Fibres and Medicinal Plants—National Research Institute, Wojska Polskiego 71b, 60-630 Poznań, Poland; marcin.ozarowski@iwnirz.pl; 4 The Neurotox Lab, School of Science, RMIT University, Plenty Road, P.O. Box 71, Bundoora, VIC 3083, Australia; donald.wlodkowic@rmit.edu.au; 5 Department of Dental Surgery, Periodontology and Oral Mucosa Diseases, Poznań University of Medical Sciences, Bukowska 70, 60-812 Poznań, Poland; marzena.wyganowska@periona.pl; 6 Division of Perinatology, Poznan University of Medical Sciences, Polna 33, 60-535 Poznań, Poland; asm@data.pl; 7 Department of Pharmacognosy and Biomaterials, Poznań University of Medical Sciences, Rokietnicka 3, 60-806 Poznań, Poland; jpiontek@ump.edu.pl

**Keywords:** antimicrobial resistance, AMR, fungi, priority pathogens, clinical concentration, clinical dose, fold change

## Abstract

**Background/Objectives:** Adaptation can reduce or completely eliminate the effectiveness of antibiotics and antiseptics at clinical concentrations. To our knowledge, no studies have examined fungal adaptation to antiseptics. This study aimed to preliminarily investigate the potential for *Candida albicans* adaptation to eight antiseptics. **Methods:** The minimal inhibitory concentration (MIC), drug susceptibility, adaptation to antiseptics, and Karpinski Adaptation Index (KAI) of *C. albicans* strains were assessed. **Results:** The antiseptics with the most effective MICs activity against *C. albicans* were octenidine dihydrochloride (OCT), chlorhexidine digluconate (CHX), and polyhexamethylene biguanide (polyhexanide, PHMB). Sodium hypochlorite (NaOCl) and ethacridine lactate (ET) demonstrated moderate activity, while boric acid (BA), povidone–iodine (PVI), and potassium permanganate (KMnO_4_) showed the weakest activity. The MIC values for NaOCl and KMnO_4_ were close to or equal to the clinical concentrations used in commercial products. The studied strains were susceptible to econazole, miconazole, and voriconazole. Resistance to other drugs occurred in 10–30% of the strains. Antifungal resistance remained unchanged after antiseptic adaptation testing. The lowest KAI values, indicating very low resistance risk, were observed for CHX, OCT, and PHMB. PVI and BA presented a low risk, ET a moderate risk. KMnO_4_ and NaOCl had the highest KAI values, indicating high and very high resistance risk in *Candida* yeasts. **Conclusions:** *C. albicans* strains can adapt to antiseptics to varying extents. For most antiseptics, adaptation does not significantly affect their clinical efficacy. However, due to adaptation, NaOCl and KMnO_4_ may become ineffective against *C. albicans* strains even at clinical concentrations.

## 1. Introduction

In 2022, the World Health Organization presented a study on fungal priority pathogens [1]. The study identified fungal pathogens that can cause invasive systemic infections and those exhibiting drug resistance. The highest priority group, labeled as ‘critical’, includes *Cryptococcus neoformans*, *Aspergillus fumigatus*, *Candida auris*, and *C. albicans*. Fungi of lower priority include other *Candida* species, such as *C. glabrata*, *C. tropicalis*, *C. parapsilosis*, and *C. krusei*.

*C. albicans* is part of the healthy human microbiome, colonizing the mouth, throat, gut, vagina, and skin, without causing infections in immunocompetent individuals. The oral colonization of *C. albicans* occurs in 23–49% of children [2,3] and approximately 35–63% of adults [4,5]. This yeast can lead to diseases such as mucosal and cutaneous candidiasis [6]. In critically ill and immunocompromised patients, it can cause invasive infections with high mortality rates [7]. Resistance to fluconazole and voriconazole has been observed in some *C. albicans* strains [8,9]. Additionally, *C. albicans* is one of the most significant fungal pathogens in wound infections, particularly in diabetic foot ulcers and burn wounds [9,10,11]. In chronic wounds, fungi from the *Candida* genus are present in 23–40% of cases, with *C. albicans* being the predominant species [12].

*C. albicans* has numerous virulence factors that contribute to infection development and immune evasion. It can exist in three forms: blastospores (yeast form), pseudohyphae, and hyphae. This morphological plasticity is crucial to its pathogenicity [13]. The hyphal forms are particularly invasive, capable of penetrating host tissues and producing candidalysin, a cytolytic peptide toxin that damages host cells [14]. *C. albicans* also produces enzymes such as proteases, phospholipases, lipases, and hemolysins, which enable host cell invasion and help evade the host’s immune response [15]. Additionally, *C. albicans* strains can form biofilms that protect them from external threats, including antifungal drugs, antiseptics, and the host immune response [16]. Biofilms pose a significant clinical challenge, especially in chronic wound infections [17].

We are on the threshold of an antibiotic crisis, with WHO data indicating a dramatic increase in deaths due to infections from multi-resistant pathogens [18,19]. Simultaneously, a growing number of fungal species are also becoming multi-resistant, reducing available therapeutic options [1,20]. Many antiseptics are currently not recommended, mainly due to their cytotoxic and allergenic effects or insufficient antimicrobial activity [21]. Increasingly, bacterial strains are reported as resistant or less sensitive to antiseptics, including chlorhexidine gluconate [22], benzalkonium chloride [22,23], cetylpyridinium chloride [24], triclosan [25], hydrogen peroxide, and povidone–iodine [26,27]. This reduced susceptibility and increased resistance to disinfectants and antiseptics may eventually lead to an ‘antiseptic crisis’ in the near future [28].

In recent years, concern has grown over the threat of bacterial adaptation to antiseptics following repeated exposure [29,30]. Such adaptation can result in a decreased or even complete loss of effectiveness of antibiotics or antiseptics at clinical concentrations [31]. To our knowledge, no studies have yet investigated the development of adaptation in fungi in response to antiseptics. However, this issue is of great importance, especially given the growing threat posed by fungal pathogens [1]. Therefore, this study aimed to explore the possibility of adaptation and, indirectly, the risk of resistance development to antiseptics in *C. albicans* yeast.

## 2. Results

### 2.1. Drug Susceptibility Testing

**Figure 1:**
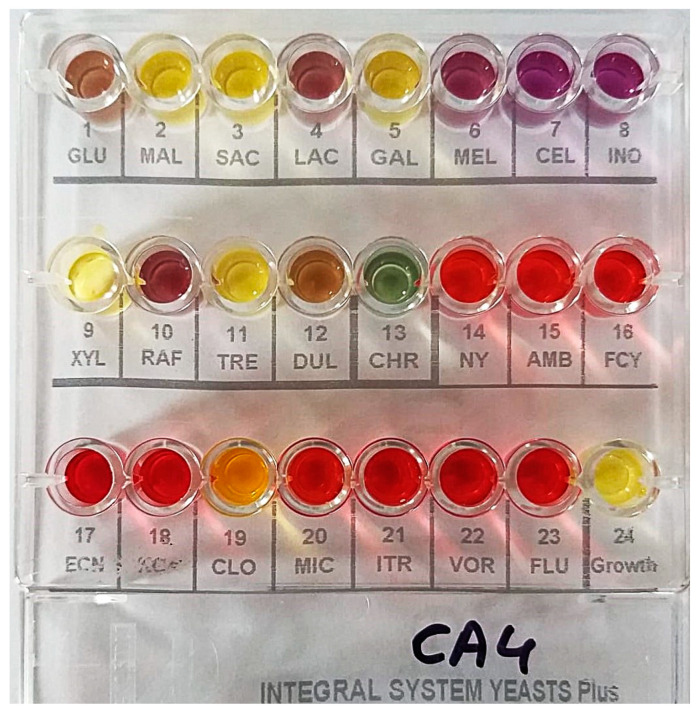
An exemplary photo of the Integral System Yeasts Plus identification test for yeast-like fungi and their drug susceptibility. In wells 1–12, a yellow-gray color indicates positive results, while purple indicates negative results. In well 13, a green color confirms the species *Candida albicans*. In wells 14–23, a red color indicates drug susceptibility (S), whereas yellow indicates drug resistance (R). A yellow color in well 24 confirms the growth of the fungus. Abbreviations: GLU Glucose, MAL Maltose, SAC Saccharose, LAC Lactose, GAL Galactose, MEL Melibiose, CEL Cellobiose, INO Inositol, XYL Xylose, RAF Raffinose, TRE Trehalose, DUL Dulcitol, CHR Chromogenic substrate, NY nystatin 1.25 µg/mL, AMB amphotericin 2 µg/mL, FCY flucytosine 16 µg/mL, ECN econazole 2 µg/mL, KCA ketoconazole 0.5 µg/mL, CLO clotrimazole 1 µg/mL, MIC miconazole 2 µg/mL, ITR itraconazole 1 µg/mL, VOR voriconazole 2 µg/mL, FLU fluconazole 4 µg/mL.

Studies revealed that all *C. albicans* strains were susceptible to econazole, miconazole, and voriconazole. Resistance was observed in 10% of the strains to fluconazole, flucytosine, and ketoconazole; in 20% of the strains to nystatin and itraconazole; and in 30% of the strains to amphotericin and clotrimazole (Figure 1, Table 1). Importantly, no changes in antifungal drug resistance were observed after adaptation testing with a specific antiseptic. This suggests that antiseptics likely do not affect changes in the drug susceptibility of *C. albicans* yeasts. Detailed raw data on the drug susceptibility of the *C. albicans* strains are presented in the supplementary file as Table S1.

### 2.2. Minimal Inhibitory Concentrations (MIC)

The studies showed that octenidine dihydrochloride (OCT), chlorhexidine digluconate (CHX), and polyhexamethylene biguanide (PHMB) exhibit the strongest activity against *C. albicans*, with minimum inhibitory concentrations (MIC) in control tests being in the range of several µg/mL. Sodium hypochlorite (NaOCl) and ethacridine lactate (ET) demonstrated weaker activity, with MICs of 100 µg/mL and 119 µg/mL, respectively. The weakest antifungal activity was observed with boric acid (BA), povidone–iodine (PVI), and potassium permanganate (KMnO₄), showing MIC values of several mg/mL. Additionally, it was found that the MIC values obtained for NaOCl and KMnO_4_ are close to or equal to the concentrations used in commercial products, namely, 100 µg/mL and 10 mg/mL, respectively.

**Figure 2:**
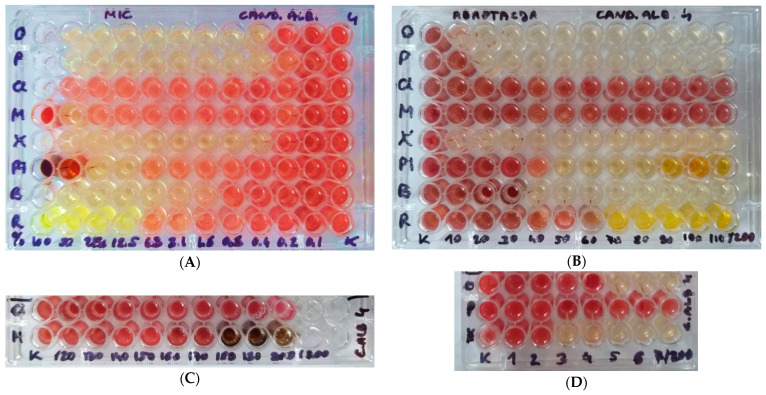
Exemplary images of plates for *Candida albicans* strain number 4. (**A**). Minimal inhibitory concentration (MIC) study; 100% concentration represents the commercial concentration of the compound, as shown in Section 2.1. (**B**). Adaptation study under the influence of increasing concentrations of antiseptics. The doses of antiseptics ranged from 10/200, i.e., 5% of the commercial concentration, to 110/200, i.e., 55% of the commercial concentration. The concentrations were increased by an additional 5% every 2 days. (**C**). Further adaptation study under the influence of NaOCl and KMnO_4_. The doses of antiseptics ranged from 120/200, i.e., 60% of the commercial concentration, to 200/200, i.e., 100% of the commercial concentration. (**D**). Additional adaptation study for OCT, PHMB, and CHX at low doses, from 1/200, i.e., 0.5%, to 7/200, i.e., 3.5%. TTC reagent was used on the plates, which turns red in the presence of microbial growth. Abbreviations: O—octenidine dihydrochloride; P—polyhexamethylene biguanide; Cl—sodium hypochlorite; M—potassium permanganate; X—chlorhexidine digluconate; PI—povidone–iodine; B—boric acid; R—ethacridine lactate.

The study presented no significant changes in most MIC levels for OCT, PHMB, NaOCl, KMnO₄, and PVI after adaptation with antiseptics. Adaptation with KMnO₄ resulted in a significant increase in MIC values for PHMB, and adaptation with NaOCl and KMnO₄ led to increased MIC values for BA. However, these increases were only about two-fold. The most MIC increases following adaptation were observed for CHX and ET. This indicates that the use of different antiseptics may lead to an approximately two-fold reduction in *C. albicans* sensitivity, particularly to CHX and ET (Table 2). Figure 2 shows the example results of the minimum inhibitory concentration (MIC) testing and adaptation study. Detailed raw data on MIC values are presented in the supplementary file as Tables S1–S9.

### 2.3. Adaptation of Candida albicans Strains to Antiseptics

The studies demonstrated that *C. albicans* yeasts undergo adaptation when exposed to increasing concentrations of antiseptics. The strains showed weak adaptation to OCT, CHX, PVI, and ET, managing to grow at concentrations several times higher than their respective MIC values. For NaOCl and KMnO₄, adaptation levels were also low but were close to or equal to the initial commercial concentration. This finding suggests that the development of adaptation may lead to the ineffectiveness of commercial products containing NaOCl and KMnO_4_ against *C. albicans*. The highest increase in adaptation relative to MIC was observed for BA and PHMB; however, these values were still much lower than the commercial concentrations.

Based on the Karpinski Adaptation Index, the lowest KAI values were observed for CHX, OCT, and PHMB, indicating a very low risk of clinical resistance development in the studied *C. albicans* strains. PVI and BA had a low risk, while ET posed a moderate risk for clinical resistance development. The highest KAI values were observed for KMnO₄ and NaOCl, indicating a high and very high risk, respectively, of clinical resistance development in *Candida* yeasts. The results of *C. albicans* adaptation to antiseptics are provided in Table 3. Detailed raw data on the *C. albicans* adaptation are presented in the supplementary file as Table S10.

## 3. Discussion

In this study, we investigated the activity of antiseptics against *C. albicans* and their potential for adaptation. Previous studies have shown similar activity for OCT [6,32,33] and CHX [6,32,33,34,35] against *C. albicans,* generally in the range of approximately 0.5 µg/mL to several µg/mL. MIC values for PHMB vary more widely, from a few µg/mL [32,33] to as high as 47 µg/mL [6].

For PVI and ET, the MIC levels observed in this study were higher than those reported in other research [32,33,35,36,37]. In contrast, NaOCl and BA have been reported with both much lower [32,34,38] and much higher MIC levels [39,40,41], with some NaOCl values reaching up to 30 times the levels found here. These findings suggest that NaOCl preparations may not be active at clinical concentrations, which we have confirmed.

No studies regarding the MIC of KMnO_4_ against *C. albicans* were found. However, for other microorganisms, KMnO₄ has shown inhibitory concentrations ranging from 300–10,000 µg/mL for *Staphylococcus aureus* [42] to 40,000 µg/mL for *Rhodotorula mucilaginosa* [43], which, similarly to NaOCl, indicates potential inactivity at clinical concentrations. The presented data are summarized in Table 4.

In the studies described, all *C. albicans* strains were found to be sensitive to econazole and miconazole. These results are consistent with data from a meta-analysis from Iran, which also reported no resistance to econazole and miconazole [45]. While no resistance to voriconazole was observed in this study, other reports suggest that resistance remains relatively uncommon, estimated at around 5–6% [45,46].

Resistance to the remaining antifungal agents was observed. Resistance to fluconazole, flucytosine, and ketoconazole each occurred in one strain (10%). Previous studies have reported varying resistance rates, with fluconazole resistance from 9% to 71%, flucytosine resistance about 42% [45,47], and ketoconazole resistance ranging from 25% to 47% in *C. albicans* [45,46,47]. Studies from Mexico indicate even higher resistance rates, with up to 95% of strains resistant to fluconazole and 97% to ketoconazole [48]. Resistance to nystatin and itraconazole was observed in 20% of strains. Interestingly, other studies report a range of resistance levels for these drugs, from 0% to 41% for nystatin and from 7% to 59% for itraconazole [45,46,47,48]. The highest resistance in this study was found for amphotericin and clotrimazole, with 30% of strains exhibiting resistance. Previous studies have reported lower resistance to amphotericin, around 9% of isolates [45], and higher resistance for clotrimazole, at levels of 45–62% [45,47]. The observed differences between these findings and those in other publications may be due to factors such as the small sample size or geographical variations in resistance prevalence.

There are several possible mechanisms leading to increased tolerance or resistance in fungi. Antiseptics generally act by disrupting cell membranes, denaturing proteins, inactivating enzymes, and causing metabolic disorders [49]. However, prolonged exposure to low or gradually increasing doses might induce additional changes, similar to those seen with antifungal drugs. For example, in fluconazole-resistant isolates, an increased thickness of ergosterol and chitin layers has been observed, which impedes drug penetration into cells. Additionally, an increase in mutations in specific genes has been noted [50]. Cross-resistance to fluconazole and voriconazole was also observed in *C. albicans* and *C. parapsilosis* strains exposed to methotrexate. In this case, changes occurred in the expression of genes in various signaling pathways [51]. The development of cross-resistance to antibacterial drugs has been demonstrated, for instance, for hypochlorites and CHX [52,53]. In this study, no cross-resistance was found between antiseptics and antifungal drugs. It is possible that a different mechanism of action of antiseptics than that of antifungal drugs is responsible for the lack of this relationship in fungi. Other studies have shown that *C. albicans* can adapt under stress conditions, involving mechanisms such as phosphatase calcineurin, the protein kinase C cell wall integrity pathway, and the molecular chaperone heat shock protein 90 [54]. Another key factor in resistance development is biofilm formation. Components like β-glucan and extracellular DNA within the biofilm matrix contribute to its resistance against antifungals, potentially increasing antifungal resistance by up to 1000-fold [55]. Additionally, as biofilm matures, the expression of efflux pump genes rises, leading to the active removal of drugs from fungal cells [56].

To our knowledge, this study is the first to examine antiseptic adaptation in *C. albicans*. While there is research on the adaptation of various antiseptic compounds against bacteria, studies on fungi, especially yeasts, are lacking. Thus, our findings can only be compared with bacterial adaptation data.

For instance, research on *Escherichia coli* found no adaptation to CHX, OCT, or NaOCl. Following adaptation procedures, the MIC values remained at 1–2 μg/mL for CHX, 2 μg/mL for OCT, and 256–512 μg/mL for NaOCl. Notably, only NaOCl showed a twofold increase in MIC_95_ values [57]. Interestingly, the MIC level for NaOCl is higher than the typical commercial concentration (100 μg/mL) used in wound treatment.

Shepherd et al. demonstrated that seven *Pseudomonas aeruginosa* strains could develop adaptive growth in response to OCT and CHX [58]. The initial concentration of OCT was 2 µg/mL. All strains developed octenidine tolerance; however, four of them were able to grow at a final concentration of 64 μg/mL. Compared to parental strains, tolerance increased 8- to 32-fold after several days of exposure to OCT. However, this adaptation level is still well below the commercial OCT concentration of 500–1000 μg/mL. *P. aeruginosa* also exhibited adaptation to CHX, with growth at final concentrations of 128–512 μg/mL, reflecting a 2- to 16-fold increase relative to the parental strains [58]. Furthermore, another study indicated that *Pseudomonas aeruginosa* adaptation to OCT is associated with mutations in efflux pump genes smvAR and phosphatidylserine synthase pssA, and occasionally in phosphatidylglycerophosphate synthase pgsA genes [59].

Increased tolerance to antiseptics was also demonstrated in another Gram-negative bacterium, *Proteus mirabilis* [60]. In studies involving only three clinical isolates, tolerance levels were found to reach 512 µg/mL for CHX and 128 µg/mL for OCT, with initial concentrations at 8 µg/mL for CHX and 2 µg/mL for OCT. This indicates a significant tolerance development, up to 64 times higher than the initial values. The authors noted that mutations resulting in the inactivation of the smvR repressor and increased expression of the smvA efflux pump also occurred in *P. mirabilis*.

When comparing these findings, it is evident that *C. albicans* yeasts exhibit weaker adaptation to increasing antiseptic concentrations than bacteria. In the case of NaOCl and KMnO_4_, the level of adaptation was similar to or equal to the clinical concentrations of commercial products, suggesting that the development of adaptation may result in reduced effectiveness of these compounds against *C. albicans*.

This study is the first to utilize the Karpinski Adaptation Index, revealing that CHX, OCT, and PHMB are antiseptics associated with a very low risk of developing clinical resistance in *C. albicans*. Conversely, a high and very high risk of clinical resistance development in *Candida* yeasts was noted for KMnO_4_ and NaOCl.

## 4. Materials and Methods

### 4.1. Antiseptics

Eight antiseptics were examined in the study: octenidine dihydrochloride (OCT; Schülke & Mayr GmbH, Norderstedt, Germany), polyhexamethylene biguanide (PHMB, Cosmocil PG; Arxada AG, Basel, Switzerland), sodium hypochlorite (NaOCl; Cerkamed, Stalowa Wola, Poland), potassium permanganate (KMnO_4_; Hasco-Lek S.A., Wrocław, Poland), chlorhexidine digluconate (CHX; Sigma-Aldrich, Poznań, Poland), povidone–iodine (PVI, poly(vinylpyrrolidone)–iodine complex; Sigma-Aldrich, Poznań, Poland), boric acid (BA, Borasol; Herbapol, Poznań, Poland), and ethacridine lactate (ET, Rivanol; Herbapol, Poznań, Poland). The initial concentrations of active substances matched the clinical concentrations found in commercial products used for wound and oral antiseptics: 0.05% (500 µg/mL) OCT, 0.1% (1000 µg/mL) PHMB, 0.01% (100 µg/mL) NaOCl, 1% (10 mg/mL) KMnO_4_, 0.1% (1000 µg/mL) CHX, 7.5% (75 mg/mL) PVI, 3% (30 mg/mL) BA, and 0.1% (1000 µg/mL) ET.

### 4.2. Candida albicans Strains

In the study, ten *C. albicans* strains were used, each isolated from routine diagnostic samples taken from patients with skin or oral fungal infections. Strains 1–5 were obtained from wounds, and strains 6–10 were isolated from cases of oral candidiasis. Specimens were collected from infected sites, such as skin lesions or oral mucosa, using sterile swabs to prevent contamination. The samples were inoculated onto CHROMagar *Candida* (Graso Biotech, Starogard Gdański, Poland) within 10 min of collection. The plates were incubated at 37 °C for 48 h to allow yeast growth. Following incubation, green colonies suggesting *C. albicans* were selected for further analysis. The yeasts were identified and confirmed using the Integral System Yeasts Plus (Liofilchem Diagnostici, Roseto, Italy). In further studies, *C. albicans* strains were cultured on Sabouraud agar or in Sabouraud broth (Graso Biotech, Starogard Gdański, Poland).

### 4.3. Drug Susceptibility Testing

Drug susceptibility testing for antifungal agents was performed using the Integral System Yeasts Plus (Liofilchem Diagnostici, Roseto, Italy). The kit contains the following concentrations: nystatin at 1.25 µg/mL, amphotericin at 2 µg/mL, flucytosine at 16 µg/mL, econazole at 2 µg/mL, ketoconazole at 0.5 µg/mL, clotrimazole at 1 µg/mL, miconazole at 2 µg/mL, itraconazole at 1 µg/mL, voriconazole at 2 µg/mL, and fluconazole at 4 µg/mL.

### 4.4. Minimal Inhibitory Concentrations (MIC)

The minimal inhibitory concentrations (MIC) of antiseptics were determined using the micro-dilution method with 96-well plates (Nest Scientific Biotechnology, Wuxi, China) with Sabouraud broth (Graso Biotech, Starogard Gdański, Poland). This method is detailed in our previous publications [6,61,62]. Each well contained a final volume of 200 µL. Serial dilutions of each antiseptic started at the concentrations specified in Section 2.1. The plates were incubated at 36 °C for 24 h. After incubation, MIC values were determined visually. In some samples, a colorimetric reaction was further enhanced by adding 10 µL of a 1% aqueous solution of 2,3,5-triphenyl-tetrazolium chloride (TTC; Sigma Aldrich, Poznań, Poland).

### 4.5. Adaptation of Candida albicans Strains to Antiseptics

**Figure 3:**
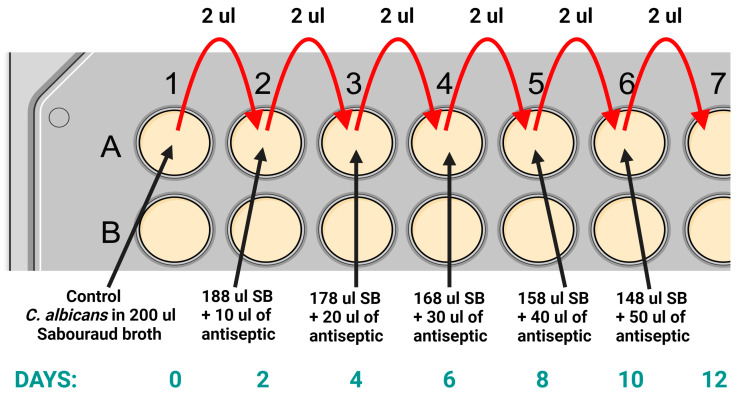
The initial stages of the study on the adaptation of *Candida albicans* strains to antiseptics were conducted on a 96-well plate. The control test was carried out in 200 µL of Sabouraud broth (SB). Every two days, 2 µL of the culture was transferred to the next well with a higher concentration of antiseptic.

*C. albicans* strains were cultured at an initial concentration of 5% of the starting concentration, which corresponded to 25 µg/mL for OCT, 50 µg/mL for PHMB, 5 µg/mL for NaOCl, 500 µg/mL for KMnO₄, 50 µg/mL for CHX, 3.75 mg/mL for PVI, 1.5 mg/mL for BA, and 50 µg/mL for ET. Additionally, for OCT, PHMB, and CHX, testing was conducted at concentrations ranging from 0.5% to 4.5% of the starting concentration, specifically, 2.5 to 22.5 µg/mL for OCT, 5 to 45 µg/mL for PHMB, and 5 to 45 µg/mL for CHX. The tests were performed in 96-well plates containing 200 µL of Sabouraud broth. Every two days, 2 µL of each culture were transferred to a fresh well with Sabouraud broth and a higher concentration of antiseptic. The study was continued until the clinical/commercial starting concentration was achieved (Figure 3). Finally, the yeasts were passaged twice without the presence of the antiseptic. On these passaged colonies, further tests were performed, including re-testing of MIC and re-testing of drug resistance.

The fold change in antiseptic adaptation relative to MIC, presented in Table 3, was calculated as the ratio of the adaptation level for a given strain to its MIC value after adaptation for the specific antiseptic. Calculations were performed on raw data, which are presented in the supplementary file.

### 4.6. Karpinski Adaptation Index (KAI)

To analyze the adaptation results and determine the potential for developing resistance to antiseptics, the Karpinski Adaptation Index (KAI) [31] was applied, according to the formula:

$$Adaptation\;Index\;(KAI)=\frac{Adaptation\;}{Clinical\;concentration}$$

where ‘Adaptation’ refers to the maximum concentration of a drug at which a microorganism can continue to grow, whereas ‘Clinical concentration’ denotes the standard commercial concentration of the drug, the clinical dosage used in treatment of a specific disease, or the recommended dose for a natural-origin compound. The Karpinski Adaptation Index (KAI) provides insights into the level of adaptation compared to the clinical concentration of commercial products, allowing for an assessment of the risk of developing clinical resistance. The results were interpreted as follows:

- KAI ≤ 0.1: Very low risk of clinical resistance. The level of adaptation is significantly lower than the clinical concentration, making the risk of resistance development very unlikely.
- 0.1 < KAI < 0.2: Low risk of clinical resistance.
- 0.2 < KAI < 0.8: Moderate risk of clinical resistance.
- 0.8 < KAI < 1.0: High risk of clinical resistance.
- KAI ≥ 1.0: Very high risk of clinical resistance. The level of adaptation is equal to or higher than the clinical concentration, potentially leading to the development of clinical resistance or indicating that resistance may have already occurred.

### 4.7. Statistics

The one-way ANOVA with Tukey post hoc tests was applied to determine the statistical significance of differences in the MIC values. Results were considered significant at the level of *p* < 0.05. Data analysis was tested using InStat3 software (GraphPad Software, Boston, MA, USA).

## 5. Conclusions

*Candida albicans* strains can adapt to antiseptics to varying extents. However, for most antiseptics, this adaptation does not significantly compromise their clinical efficacy. In the cases of NaOCl and KMnO_4_, adaptation levels were comparable to the clinical concentrations of the commercial formulations used in wound care and oral infections. Consequently, this suggests that NaOCl and KMnO_4_ may be ineffective against *C. albicans* strains even at clinically relevant concentrations. The application of the Karpinski Adaptation Index (KAI) is significant in evaluating the risk of resistance development to clinical concentrations of medicines.

## Figures and Tables

**Table 1:** Drug susceptibility of the tested *Candida albicans* strains to antifungal drugs.

Antifungal Drug	Percentage of Sensitive (S) *C. albicans* Strains	Percentage of Resistant (R) *C. albicans* Strains
ECN	100%	0%
MIC	100%	0%
VOR	100%	0%
AMB	90%	10%
KCA	90%	10%
FLU	90%	10%
ITR	80%	20%
NY	80%	20%
CLO	70%	30%
FCY	70%	30%

Abbreviations: S—sensitivity; R—resistance; ECN—econazole 2 µg/mL; MIC—miconazole 2 µg/mL; VOR—voriconazole 2 µg/mL; AMB—amphotericin 2 µg/mL; KCA—ketoconazole 0.5 µg/mL; FLU—fluconazole 4 µg/mL; ITR—itraconazole 1 µg/mL; NY—nystatin 1.25 µg/mL; CLO—clotrimazole 1 µg/mL; FCY—flucytosine 16 µg/mL.

**Table 2:** Minimum inhibitory concentration (MIC) values for control samples before adaptation and for samples after adaptation with a specific antiseptic against 10 *Candida albicans* strains.

Antiseptic [Units]	Control MIC Before Adaptation	MIC Values (Mean ± SD) After Adaptation with Below Antiseptic							
		OCT	PHMB	NaOCl	KMnO_4_	CHX	PVI	BA	ET
OCT [µg/mL]	2.73 ± 0.98	3.32 ± 0.92	3.12 ± 0.98	3.32 ± 0.92	3.32 ± 0.92	3.02 ± 1.0	3.02 ± 1.0	3.22 ± 0.95	3.02 ± 1.0
PHMB [µg/mL]	4.01 ± 1.78	4.29 ± 1.63	5.27 ± 1.91	4.58 ± 2.03	6.92 ± 4.88 **	5.07 ± 2.14	5.27 ± 3.96	4.58 ± 2.03	4.49 ± 2.11
NaOCl [µg/mL]	100 ± 0.0	100 ± 0.0	110 ± 30.8	100 ± 0.0	100 ± 0.0	100 ± 0.0	100 ± 0.0	100 ± 0.0	100 ± 0.0
KMnO_4_ [mg/mL]	8.5 ± 2.35	9.0 ± 2.05	9.0 ± 2.05	10.0 ± 3.97	10.0 ± 3.97	10.0 ± 3.97	9.0 ± 2.05	9.0 ± 2.05	9.0 ± 2.05
CHX [µg/mL]	3.61 ± 0.71	5.17 ± 2.31 *	5.75 ± 3.26 ***	5.75 ± 3.26 ***	4.78 ± 2.14	4.88 ± 2.05	4.78 ± 2.14	5.75 ± 3.26 ***	4.78 ± 2.14
PVI [mg/mL]	8.44 ± 1.93	8.91 ± 1.44	8.91 ± 1.44	8.91 ± 1.44	8.91 ± 1.44	8.91 ± 1.44	8.91 ± 1.44	8.91 ± 1.44	8.91 ± 1.44
BA [mg/mL]	1.62 ± 0.88	1.99 ± 1.25	1.99 ± 1.25	2.93 ± 2.59 ***	2.93 ± 2.59 ***	2.02 ± 1.23	1.99 ± 1.25	2.02 ± 1.23	1.99 ± 1.25
ET [µg/mL]	118.8 ± 63.8	243.8 ± 181.3 ***	243.8 ± 181.3 ***	243.8 ± 181.3 ***	243.8 ± 181.3 ***	243.8 ± 181.3 ***	243.8 ± 181.3 ***	243.8 ± 181.3 ***	246.9 ± 178.5 ***

Statistically significant differences compared to the control before adaptation: * *p* < 0.05, ** *p* < 0.01, *** *p* < 0.001. Abbreviations: OCT—octenidine dihydrochloride; PHMB—polyhexamethylene biguanide; NaOCl—sodium hypochlorite; KMnO_4_—potassium permanganate; CHX—chlorhexidine digluconate; PVI—povidone–iodine; BA—boric acid; ET—ethacridine lactate.

**Table 3:** Adaptation of *Candida albicans* strains to antiseptics. The highest concentrations of antiseptics at which yeast growth was observed are presented.

Antiseptic	Mean Adaptation ± SD	Adaptation Fold Change Relative to MIC	Karpinski Adaptation Index (KAI)
OCT [µg/mL]	9.5 ± 1.05	×1.9–5.1	0.019
PHMB [µg/mL]	71 ± 3.16	×4.5–35.9	0.071
NaOCl [µg/mL]	100 ± 0.0	×1.0	1.0
KMnO_4_ [mg/mL]	8.55 ± 0.16	×0.4–1.7	0.855
CHX [µg/mL]	8.0 ± 2.58	×0.6–2.6	0.008
PVI [mg/mL]	13.5 ± 1.94	×1.2–2.4	0.18
BA [mg/mL]	4.5 ± 0.0	×0.6–9.6	0.15
ET [µg/mL]	260 ± 31.6	×0.5–4.0	0.26

Interpretation of the Karpinski Adaptation Index (KAI): KAI ≤ 0.1: very low risk of clinical resistance; 0.1 < KAI < 0.2: low risk of clinical resistance; 0.2 < KAI < 0.8: moderate risk of clinical resistance; 0.8 < KAI < 1.0: high risk of clinical resistance; KAI ≥ 1.0: very high risk of clinical resistance [31]. Abbreviations: OCT—octenidine dihydrochloride; PHMB—polyhexamethylene biguanide; NaOCl—sodium hypochlorite; KMnO_4_—potassium permanganate; CHX—chlorhexidine digluconate; PVI—povidone–iodine; BA—boric acid; ET—ethacridine lactate.

**Table 4:** Comparison of the results of minimal inhibitory concentrations (MIC) of antiseptics against *Candida albicans* obtained in this study and values presented in the literature.

Antiseptic	Mean control MICs Obtained in This Study (µg/mL)	Mean MICs in the Literature (All Values Were Converted to µg/mL)
Octenidine dihydrochloride	2.73	0.5–0.9 [6], 0.5–1.0 [32], 1.0 [33]
Polyhexamethylene biguanide	4.01	1 [33], 3.9–7.8 [32], 11.7–46.9 [6]
Sodium hypochlorite	100	<10 [34], no activity up to 80 [32], 3000 [39], 3300 [40],
Potassium permanganate	8500	300–10,000 for *Staphylococcus aureus* [42], 40,000 for *Rhodotorula mucilaginosa* [43]
Chlorhexidine digluconate	3.61	<0.63 [34], 1.1–2.4 [6], 2.45–4.9 [32], 4.0 [33], 0.5–16 [35]
Povidone–iodine	8440	70–250 [35], 256 [33], 1170–2348 [32], 4000 [36]
Boric acid	1620	0.82–52.5 [38], 940–1870 [44] 1563–6250 [41]
Ethacridine lactate	118.8	<1.0 [37], 31.3–62.5 [32]

## Data Availability

The original contributions presented in the study are included in the article, and further inquiries can be directed to the corresponding author.

## Supplementary Materials

The following supporting information can be downloaded at: https://www.mdpi.com/article/10.3390/ph17111544/s1. **Table S1.** Drug susceptibility of the tested *Candida albicans* strains to antifungal drugs. **Table S2.** Minimum inhibitory concentration (MIC) values for control samples before adaptation and for samples after adaptation with OCT against 10 *C. albicans* strains. **Table S3.** Minimum inhibitory concentration (MIC) values for control samples before adaptation and for samples after adaptation with PHMB against 10 *C. albicans* strains. **Table S4.** Minimum inhibitory concentration (MIC) values for control samples before adaptation and for samples after adaptation with NaOCl against 10 *C. albicans* strains. **Table S5.** Minimum inhibitory concentration (MIC) values for control samples before adaptation and for samples after adaptation with KMnO4 against 10 *C. albicans* strains. **Table S6.** Minimum inhibitory concentration (MIC) values for control samples before adaptation and for samples after adaptation with CHX against 10 *C. albicans* strains. **Table S7.** Minimum inhibitory concentration (MIC) values for control samples before adaptation and for samples after adaptation with PVI against 10 *C. albicans* strains. **Table S8.** Minimum inhibitory concentration (MIC) values for control samples before adaptation and for samples after adaptation with BA against 10 *C. albicans* strains. **Table S9.** Minimum inhibitory concentration (MIC) values for control samples before adaptation and for samples after adaptation with ET against 10 *C. albicans* strains. **Table S10.** Adaptation of *C. albicans* strains to antiseptics. The highest concentrations of antiseptics at which yeast growth was observed are shown.
