# Supplementary material for: Adaptation to Sodium Hypochlorite and Potassium Permanganate May Lead to Their Ineffectiveness Against Candida albicans
Source: Pharmaceuticals (Basel). 2024 Nov 17;17(11):1544. doi: 10.3390/ph17111544 (PMC11597340; doi:10.3390/ph17111544)
Supplement: Supplementary file 1 [file pharmaceuticals-17-01544-s001.zip › pharmaceuticals-3306067-supplementary.pdf]

SUPPLEMENTARY MATERIALS:

Adaptation to sodium hypochlorite and potassium permanganate may lead to their ineffectiveness against *Candida albicans*

Tomasz M. Karpiński, Marzena Korbecka-Paczkowska, Marcin Ożarowski, Donald Włodkowic, Marzena Liliana Wyganowska, Agnieszka Seremak-Mrozikiewicz and Judyta Cielecka-Piontek

Abbreviations:
S - sensitivity, R - resistance;
ECN - econazole 2 µg/mL, MIC - miconazole 2 µg/mL, VOR - voriconazole 2 µg/mL, AMB - amphotericin 2 µg/mL, KCA - ketoconazole 0.5 µg/mL, FLU - fluconazole 4 µg/mL, ITR - itraconazole 1 µg/mL, NY - nystatin 1.25 µg/mL, CLO - clotrimazole 1 µg/mL, and FCY - flucytosine 16 µg/mL;
OCT - octenidine dihydrochloride, PHMB - polyhexamethylene biguanide, NaOCl - sodium hypochlorite, KMnO4 - potassium permanganate, CHX - chlorhexidine digluconate, PVI - povidone-iodine, BA - boric acid, and ET - ethacridine lactate.

Table S1. Drug susceptibility of the tested *Candida albicans* strains to antifungal drugs.

**Antifungal drug**	***Candida albicans* strain**									
	**1**	**2**	**3**	**4**	**5**	**6**	**7**	**8**	**9**	**10**
ECN	S	S	S	S	S	S	S	S	S	S
MIC	S	S	S	S	S	S	S	S	S	S
VOR	S	S	S	S	S	S	S	S	S	S
AMB	S	S	S	S	S	S	R	S	S	S
KCA	S	S	S	S	S	S	R	S	S	S
FLU	S	S	S	S	S	S	R	S	S	S
NY	S	S	S	S	R	S	S	S	R	S
ITR	S	S	S	S	S	S	R	R	S	S
CLO	S	S	S	R	S	S	R	S	S	R
FCY	S	S	S	S	R	S	R	S	R	S

Table S2. Minimum inhibitory concentration (MIC) values [μg/ml] for control samples before adaptation and for samples after adaptation with OCT against 10 *Candida albicans* strains.

C. albicans strain number	Control OCT MIC before adaptation	MIC values (mean ± SD) after adaptation with below antiseptic							
		OCT	PHMB	NaOCl	KMnO4	CHX	PVI	BA	ET
1	3.90	1.95	3.90	1.95	3.90	1.95	1.95	1.95	1.95
	1.95	1.95	1.95	1.95	3.90	1.95	1.95	3.90	1.95
2	1.95	3.90	3.90	3.90	3.90	1.95	3.90	1.95	1.95
	1.95	3.90	3.90	3.90	3.90	3.90	3.90	1.95	3.90
3	1.95	1.95	1.95	3.90	1.95	1.95	1.95	3.90	3.90
	1.95	1.95	1.95	3.90	1.95	3.90	1.95	3.90	1.95
4	3.90	3.90	3.90	3.90	3.90	3.90	3.90	3.90	3.90
	3.90	3.90	3.90	3.90	3.90	3.90	3.90	3.90	3.90
5	1.95	1.95	1.95	3.90	1.95	1.95	3.90	1.95	1.95
	1.95	1.95	1.95	3.90	1.95	1.95	3.90	1.95	3.90
6	1.95	3.90	1.95	1.95	1.95	1.95	1.95	1.95	1.95
	3.90	3.90	3.90	3.90	3.90	3.90	3.90	3.90	3.90
7	1.95	3.90	3.90	1.95	1.95	3.90	1.95	1.95	1.95
	1.95	3.90	3.90	3.90	3.90	3.90	1.95	3.90	3.90
8	1.95	3.90	1.95	1.95	3.90	1.95	1.95	3.90	1.95
	3.90	3.90	3.90	3.90	3.90	3.90	3.90	3.90	3.90
9	1.95	3.90	1.95	1.95	3.90	1.95	1.95	3.90	1.95
	3.90	3.90	3.90	3.90	3.90	3.90	3.90	3.90	3.90
10	3.90	3.90	3.90	3.90	3.90	3.90	3.90	3.90	3.90
	3.90	3.90	3.90	3.90	3.90	3.90	3.90	3.90	3.90

Table S3. Minimum inhibitory concentration (MIC) values [μg/ml] for control samples before adaptation and for samples after adaptation with PHMB against 10 *Candida albicans* strains.

C. albicans strain number	Control PHMB MIC before adaptation	MIC values (mean ± SD) after adaptation with below antiseptic							
		OCT	PHMB	NaOCl	KMnO4	CHX	PVI	BA	ET
1	7.80	7.80	7.80	7.80	7.80	7.80	7.80	7.80	7.80
	7.80	7.80	7.80	7.80	7.80	7.80	7.80	7.80	7.80
2	1.95	1.95	3.90	1.95	1.95	3.90	1.95	1.95	1.95
	1.95	3.90	3.90	3.90	1.95	1.95	1.95	3.90	1.95
3	1.95	3.90	7.80	1.95	1.95	1.95	1.95	1.95	1.95
	1.95	1.95	7.80	1.95	3.90	3.90	1.95	1.95	1.95
4	3.90	3.90	3.90	3.90	3.90	3.90	3.90	3.90	3.90
	3.90	3.90	3.90	3.90	3.90	7.80	3.90	3.90	3.90
5	3.90	3.90	7.80	7.80	15.60	7.80	15.60	7.80	3.90
	3.90	3.90	7.80	7.80	15.60	3.90	15.60	7.80	3.90
6	3.90	3.90	3.90	3.90	3.90	3.90	3.90	3.90	3.90
	3.90	3.90	3.90	3.90	7.80	7.80	3.90	3.90	7.80
7	3.90	3.90	3.90	3.90	7.80	7.80	3.90	3.90	7.80
	7.80	7.80	7.80	7.80	7.80	7.80	7.80	7.80	7.80
8	3.90	3.90	3.90	3.90	15.60	3.90	3.90	3.90	3.90
	3.90	3.90	3.90	3.90	15.60	3.90	3.90	3.90	3.90
9	3.90	3.90	3.90	3.90	3.90	3.90	3.90	3.90	3.90
	3.90	3.90	3.90	3.90	3.90	3.90	3.90	3.90	3.90
10	3.90	3.90	3.90	3.90	3.90	3.90	3.90	3.90	3.90
	3.90	3.90	3.90	3.90	3.90	3.90	3.90	3.90	3.90

Table S4. Minimum inhibitory concentration (MIC) values [µg/ml] for control samples before adaptation and for samples after adaptation with NaOCl against 10 *Candida albicans* strains.

C. albicans strain number	Control NaOCl MIC before adaptation	MIC values (mean ± SD) after adaptation with below antiseptic							
		**OCT**	**PHMB**	**NaOCl**	**KMnO4**	**CHX**	**PVI**	**BA**	**ET**
1	100	100	100	100	100	100	100	100	100
	100	100	100	100	100	100	100	100	100
2	100	100	>100	100	100	100	100	100	100
	100	100	>100	100	100	100	100	100	100
3	100	100	100	100	100	100	100	100	100
	100	100	100	100	100	100	100	100	100
4	100	100	100	100	100	100	100	100	100
	100	100	100	100	100	100	100	100	100
5	100	100	100	100	100	100	100	100	100
	100	100	100	100	100	100	100	100	100
6	100	100	100	100	100	100	100	100	100
	100	100	100	100	100	100	100	100	100
7	100	100	100	100	100	100	100	100	100
	100	100	100	100	100	100	100	100	100
8	100	100	100	100	100	100	100	100	100
	100	100	100	100	100	100	100	100	100
9	100	100	100	100	100	100	100	100	100
	100	100	100	100	100	100	100	100	100
10	100	100	100	100	100	100	100	100	100
	100	100	100	100	100	100	100	100	100

Table S5. Minimum inhibitory concentration (MIC) values [mg/ml] for control samples before adaptation and for samples after adaptation with KMnO4 against 10 *Candida albicans* strains.

C. albicans strain number	Control KMnO4 MIC before adaptation	MIC values (mean ± SD) after adaptation with below antiseptic							
		**OCT**	**PHMB**	**NaOCl**	**KMnO4**	**CHX**	**PVI**	**BA**	**ET**
1	10	10	10	10	10	10	10	10	10
	10	10	10	10	10	10	10	10	10
2	5	5	5	5	5	5	5	5	5
	5	5	5	5	5	5	5	5	5
3	10	10	10	10	10	10	10	10	10
	10	10	10	10	10	10	10	10	10
4	10	10	10	20	20	20	10	10	10
	10	10	10	20	20	20	10	10	10
5	5	10	10	10	10	10	10	10	10
	5	10	10	10	10	10	10	10	10
6	10	10	10	10	10	10	10	10	10
	10	10	10	10	10	10	10	10	10
7	10	10	10	10	10	10	10	10	10
	10	10	10	10	10	10	10	10	10
8	10	10	10	10	10	10	10	10	10
	10	10	10	10	10	10	10	10	10
9	5	5	5	5	5	5	5	5	5
	5	5	5	5	5	5	5	5	5
10	10	10	10	10	10	10	10	10	10
	10	10	10	10	10	10	10	10	10

Table S6. Minimum inhibitory concentration (MIC) values [μg/ml] for control samples before adaptation and for samples after adaptation with CHX against 10 *Candida albicans* strains.

C. albicans strain number	Control CHX MIC before adaptation	MIC values (mean ± SD) after adaptation with below antiseptic							
		**OCT**	**PHMB**	**NaOCl**	**KMnO4**	**CHX**	**PVI**	**BA**	**ET**
1	3.90	3.90	3.90	3.90	3.90	3.90	3.90	3.90	3.90
	3.90	3.90	3.90	3.90	3.90	3.90	3.90	3.90	3.90
2	1.95	1.95	1.95	1.95	1.95	1.95	1.95	1.95	1.95
	1.95	1.95	1.95	1.95	1.95	1.95	1.95	1.95	1.95
3	1.95	1.95	1.95	1.95	1.95	3.90	1.95	1.95	1.95
	3.90	3.90	3.90	3.90	3.90	3.90	3.90	3.90	3.90
4	3.90	3.90	3.90	3.90	3.90	3.90	3.90	3.90	3.90
	3.90	7.80	7.80	7.80	7.80	7.80	7.80	7.80	7.80
5	3.90	7.80	7.80	7.80	7.80	7.80	7.80	7.80	7.80
	3.90	3.90	15.60	15.60	3.90	3.90	3.90	15.60	3.90
6	3.90	3.90	7.80	7.80	3.90	3.90	3.90	7.80	3.90
	3.90	7.80	7.80	7.80	3.90	3.90	3.90	7.80	3.90
7	3.90	7.80	3.90	3.90	3.90	3.90	3.90	3.90	3.90
	3.90	3.90	3.90	3.90	3.90	3.90	3.90	3.90	3.90
8	3.90	3.90	3.90	3.90	3.90	3.90	3.90	3.90	3.90
	3.90	3.90	3.90	3.90	3.90	3.90	3.90	3.90	3.90
9	3.90	7.80	7.80	7.80	7.80	7.80	7.80	7.80	7.80
	3.90	7.80	7.80	7.80	7.80	7.80	7.80	7.80	7.80
10	3.90	7.80	7.80	7.80	7.80	7.80	7.80	7.80	7.80
	3.90	7.80	7.80	7.80	7.80	7.80	7.80	7.80	7.80

Table S7. Minimum inhibitory concentration (MIC) values [mg/ml] for control samples before adaptation and for samples after adaptation with PVI against 10 *Candida albicans* strains.

C. albicans strain number	Control PVI MIC before adaptation	MIC values (mean ± SD) after adaptation with below antiseptic							
		**OCT**	**PHMB**	**NaOCl**	**KMnO4**	**CHX**	**PVI**	**BA**	**ET**
1	9.38	9.38	9.38	9.38	9.38	9.38	9.38	9.38	9.38
	9.38	9.38	9.38	9.38	9.38	9.38	9.38	9.38	9.38
2	9.38	9.38	9.38	9.38	9.38	9.38	9.38	9.38	9.38
	9.38	9.38	9.38	9.38	9.38	9.38	9.38	9.38	9.38
3	4.69	4.69	4.69	4.69	4.69	4.69	4.69	4.69	4.69
	4.69	4.69	4.69	4.69	4.69	4.69	4.69	4.69	4.69
4	9.38	9.38	9.38	9.38	9.38	9.38	9.38	9.38	9.38
	9.38	9.38	9.38	9.38	9.38	9.38	9.38	9.38	9.38
5	9.38	9.38	9.38	9.38	9.38	9.38	9.38	9.38	9.38
	9.38	9.38	9.38	9.38	9.38	9.38	9.38	9.38	9.38
6	9.38	9.38	9.38	9.38	9.38	9.38	9.38	9.38	9.38
	9.38	9.38	9.38	9.38	9.38	9.38	9.38	9.38	9.38
7	9.38	9.38	9.38	9.38	9.38	9.38	9.38	9.38	9.38
	9.38	9.38	9.38	9.38	9.38	9.38	9.38	9.38	9.38
8	9.38	9.38	9.38	9.38	9.38	9.38	9.38	9.38	9.38
	9.38	9.38	9.38	9.38	9.38	9.38	9.38	9.38	9.38
9	4.69	9.38	9.38	9.38	9.38	9.38	9.38	9.38	9.38
	4.69	9.38	9.38	9.38	9.38	9.38	9.38	9.38	9.38
10	9.38	9.38	9.38	9.38	9.38	9.38	9.38	9.38	9.38
	9.38	9.38	9.38	9.38	9.38	9.38	9.38	9.38	9.38

Table S8. Minimum inhibitory concentration (MIC) values [mg/ml] for control samples before adaptation and for samples after adaptation with BA against 10 *Candida albicans* strains.

C. albicans strain number	Control BA MIC before adaptation	MIC values (mean ± SD) after adaptation with below antiseptic							
		OCT	PHMB	NaOCl	KMnO4	CHX	PVI	BA	ET
1	3.75	3.75	3.75	7.50	7.50	3.75	3.75	3.75	3.75
	3.75	3.75	3.75	7.50	7.50	3.75	3.75	3.75	3.75
2	0.94	0.94	0.94	0.94	0.94	0.94	0.94	0.94	0.94
	1.88	1.88	1.88	1.88	1.88	1.88	1.88	1.88	1.88
3	0.94	0.94	0.94	0.94	0.94	0.94	0.94	0.94	0.94
	0.94	0.94	0.94	0.94	0.94	0.94	0.94	0.94	0.94
4	0.47	0.47	0.47	0.47	0.47	0.94	0.47	0.94	0.47
	0.94	0.94	0.94	0.94	0.94	0.94	0.94	0.94	0.94
5	1.88	1.88	1.88	3.75	3.75	1.88	1.88	1.88	1.88
	1.88	1.88	1.88	3.75	3.75	1.88	1.88	1.88	1.88
6	0.94	0.94	0.94	0.94	0.94	0.94	0.94	0.94	0.94
	1.88	1.88	1.88	1.88	1.88	1.88	1.88	1.88	1.88
7	0.94	0.94	0.94	0.94	0.94	0.94	0.94	0.94	0.94
	1.88	1.88	1.88	1.88	1.88	1.88	1.88	1.88	1.88
8	0.94	0.94	0.94	0.94	0.94	0.94	0.94	0.94	0.94
	0.94	0.94	0.94	0.94	0.94	0.94	0.94	0.94	0.94
9	1.88	3.75	3.75	7.50	7.50	3.75	3.75	3.75	3.75
	1.88	3.75	3.75	7.50	7.50	3.75	3.75	3.75	3.75
10	1.88	3.75	3.75	3.75	3.75	3.75	3.75	3.75	3.75
	1.88	3.75	3.75	3.75	3.75	3.75	3.75	3.75	3.75

Table S9. Minimum inhibitory concentration (MIC) values [μg/ml] for control samples before adaptation and for samples after adaptation with ET against 10 *Candida albicans* strains.

C. albicans strain number	Control ET MIC before adaptation	MIC values (mean ± SD) after adaptation with below antiseptic							
		OCT	PHMB	NaOCl	KMnO4	CHX	PVI	BA	ET
1	62.5	125.0	125.0	125.0	125.0	125.0	125.0	125.0	125.0
	62.5	125.0	125.0	125.0	125.0	125.0	125.0	125.0	125.0
2	62.5	62.5	62.5	62.5	62.5	62.5	62.5	62.5	125.0
	62.5	62.5	62.5	62.5	62.5	62.5	62.5	62.5	62.5
3	62.5	125.0	125.0	125.0	125.0	125.0	125.0	125.0	125.0
	125.0	125.0	125.0	125.0	125.0	125.0	125.0	125.0	125.0
4	125.0	250.0	250.0	250.0	250.0	250.0	250.0	250.0	250.0
	125.0	250.0	250.0	250.0	250.0	250.0	250.0	250.0	250.0
5	250.0	500.0	500.0	500.0	500.0	500.0	500.0	500.0	500.0
	250.0	500.0	500.0	500.0	500.0	500.0	500.0	500.0	500.0
6	125.0	125.0	125.0	125.0	125.0	125.0	125.0	125.0	125.0
	125.0	125.0	125.0	125.0	125.0	125.0	125.0	125.0	125.0
7	62.5	62.5	62.5	62.5	62.5	62.5	62.5	62.5	62.5
	62.5	62.5	62.5	62.5	62.5	62.5	62.5	62.5	62.5
8	125.0	500.0	500.0	500.0	500.0	500.0	500.0	500.0	500.0
	250.0	500.0	500.0	500.0	500.0	500.0	500.0	500.0	500.0
9	125.0	250.0	500.0	500.0	500.0	500.0	250.0	500.0	500.0
	125.0	250.0	500.0	500.0	500.0	500.0	250.0	500.0	500.0
10	62.5	125.0	125.0	125.0	125.0	125.0	125.0	125.0	125.0
	125.0	250.0	250.0	250.0	250.0	250.0	250.0	250.0	250.0

Table S10. Adaptation of *Candida albicans* strains to antiseptics. The highest concentrations of antiseptics at which yeast growth was observed are shown.

Antiseptic	*Candida albicans* strain number									
	**1**	**2**	**3**	**4**	**5**	**6**	**7**	**8**	**9**	**10**
OCT	10	7.5	10	10	10	10	10	10	7.5	10
[μg/mL]	10	7.5	10	10	10	10	10	10	7.5	10
PHMB	70	70	70	70	70	70	70	80	70	70
[μg/mL]	70	70	70	70	70	70	70	80	70	70
NaOCl	100	100	100	100	100	100	100	100	100	100
[μg/mL]	100	100	100	100	100	100	100	100	100	100
$KMnO_4$	8.5	8.5	8.5	8.5	8.5	8.5	9.0	8.5	8.5	8.5
[mg/mL]	8.5	8.5	8.5	8.5	8.5	8.5	9.0	8.5	8.5	8.5
CHX	10	5	5	10	10	10	10	5	10	5
[μg/mL]	10	5	5	10	10	10	10	5	10	5
PVI	15	15	11.25	15	11.25	15	15	11.25	15	11.25
[mg/mL]	15	15	11.25	15	11.25	15	15	11.25	15	11.25
BA	4.5	4.5	4.5	4.5	4.5	4.5	4.5	4.5	4.5	4.5
[mg/mL]	4.5	4.5	4.5	4.5	4.5	4.5	4.5	4.5	4.5	4.5
ET	200	250	250	300	250	300	250	250	300	250
[μg/mL]	200	250	250	300	250	300	250	250	300	250
